# Relationships of leisure-time physical activity and work ability between different occupational physical demands in adult working men

**DOI:** 10.1007/s00420-019-01410-x

**Published:** 2019-01-31

**Authors:** Ville Päivärinne, Hannu Kautiainen, Ari Heinonen, Ilkka Kiviranta

**Affiliations:** 1 Department of Orthopaedics and Traumatology, University of Helsinki, P.O. Box 63, Helsinki, 00014 Finland; 2 Primary Health Care Unit, Kuopio University Hospital, Kuopio, Finland; 3 Folkhälsan Research Center, Helsinki, Finland; 4 Faculty of Sport and Health Sciences, University of Jyväskylä, Jyväskylä, Finland; 5 Helsinki University Hospital, Helsinki, Finland

**Keywords:** Exercise, MET, Questionnaires, Occupational physical demands, Occupational health

## Abstract

**Purpose:**

Leisure-time physical activity (LTPA) is known to be associated with positive health benefits, but the role of occupational physical demands remains inconsistent. The purpose of the current study was to assess the relationship between LTPA and work ability in different occupational physical activity (OPA) levels between young adult men.

**Methods:**

We performed physical activity measurements in work and leisure time with the long version of International Physical Activity Questionnaire (IPAQ) and work ability with the Work Ability Index (WAI) in 921 Finnish employed male volunteer participants. The participants were divided into LTPA tertiles I (< 8 MET-h/week), II (8–28 MET-h/week), and III (> 28 MET-h/week) and OPA tertiles I (0 MET-h/week), II (< 64 MET-h/week), and III (≥ 64 MET-h/week).

**Results:**

There was a significant relationship between LTPA and WAI in OPA tertiles (adjusted for age, alcohol consumption, working class status, BMI, and employment years). Moreover, each LTPA tertile showed significant linear associations with WAI (*P* < 0.001).

**Conclusion:**

LTPA is positively associated with work ability among young adult men. More specifically, the relationships between LTPA and WAI were significantly greater in physically demanding jobs than in more passive jobs. Our results indicate the importance of LTPA, particularly with individuals under higher work-related physical strain.

## Introduction

The health benefits of physical activity (PA) are well documented, even though most of the results are restricted to leisure-time physical activity (LTPA) (Warburton et al. 2006; Lear et al. 2017). On the contrary, particularly in individuals with low fitness levels, growing evidence emphasizes the role of occupational physical activity (OPA) as not only inadequate but detrimental to health, particularly among men (Li et al. 2013; Holtermann et al. 2016, 2018; Coenen et al. 2018). The vast majority of Finnish adults reportedly do not meet PA recommendations, which increases the risk for several diseases such as cardiovascular and musculoskeletal disorders (Biswas et al. 2015; Bennie et al. 2017). In addition, the imbalance of daily PA can decrease overall health and functionality with reduced work performance, while resulting in an increased risk for long-term sickness absence and premature retirement due to disability (Holtermann et al. 2012, 2016; Mänty et al. 2015; Søgaard and Sjøgaard 2017).

The concept of work ability relates to individual physical and mental capacities and the demands of the job, where it can be used as a tool by professionals for health promotion and disease and injury prevention (Tengland 2011). Previous evidence has shown that more physically active occupations, such as construction, have a higher risk of work-related disability than less physically demanding occupations (Alavinia et al. 2009). In Finland, a little less than a third of male employees consider their work as physically demanding (Kauppinen et al. 2013). In addition, work itself is insufficient to prevent decline in work ability; measures to promote work ability should start before middle age, especially among workers in physically demanding jobs (Ilmarinen et al. 1997). Work ability can be assessed by the Work Ability Index (WAI), which is a widely used and well-accepted self-reported instrument that measures health and functional capacity via a single dimension of work ability (Ilmarinen et al. 2005).

Even though the deleterious health effects of a sedentary lifestyle and the beneficial effects of PA in adult populations are currently widely acknowledged (Warburton et al. 2006; Biswas et al. 2015), further research between more specific dimensions, i.e. LTPA and WAI within domains of OPA, is warranted. Previous studies have presented a strong positive association between the lack of vigorous LTPA and poor WAI (van den Berg et al. 2009) and there has been relationship when achieving recommended LTPA levels and better WAI among sedentary workers (Nawrocka et al. 2017). In addition, previous study found an association between high LTPA levels and better work ability in middle-aged workers with physically demanding jobs (Calatayud et al. 2015). However, the evidence remains limited for an interaction between LTPA and OPA in work ability, particularly among young adult men.

The purpose of this cross-sectional, population-based study was to determine the association between LTPA and WAI in interaction with OPA dimensions in young adult men.

## Methods

### Design and participants

The participants for this cross-sectional, population-based study were collected in 2009 from five cohorts [birth years 1969 (*N* = 67), 1974 (*N* = 139), 1979 (*N* = 228), 1984 (*N* = 229), or 1989 (*N* = 258)] of Finnish men, who had performed, withdrew from, or discontinued military service or had performed an alternative non-military service. Immigrants, imprisoned subjects, those with mental disorders, or the unemployed were excluded from the study. Initially, 1425 male participants who answered for the PA questionnaire were extracted as a population sample. Informed consent was obtained from all individual participants included in the study.

### Questionnaire

A questionnaire was applied to record PA, work ability, health behavioral and functional capacity, musculoskeletal disorders, mental disorders, pain, and alcohol consumption. The questions were partly based on the Finnish Health 2000 study, the IPAQ questionnaire, and WAI questionnaire (Aromaa and Koskinen 2004; International Physical Activity Questionnaire team 2005; Rautio and Michelsen 2014).

### Physical activity

The long version of the International Physical Activity Questionnaire (IPAQ-long) in the Finnish language was used to estimate the level of LTPA and OPA. The IPAQ comprises of four different detailed PA levels (work-related activity, leisure-time activity, transport-related activity, and domestic and garden activities) that require respondents to recall their PA over the past 7 days. In our study, we concentrated to work-related activity as OPA and leisure-time activity as LTPA, wherein both domains contained three levels of intensity (walking, moderate, or vigorous) to provide domain-specific scores. Both domains of activity are defined in metabolic equivalent minutes per week (MET-min/week) by multiplying the MET value for the activity (3.3 for walking, 4.0–6.0 for moderate-intensity activity, and 8.0 for vigorous-intensity activity) with duration (minutes) and frequency (days). After this, we converted values to more broadly used MET-hours per week (MET-h/week) (International Physical Activity Questionnaire team 2005). IPAQ is a valid and reliable instrument for assessing levels and patterns of PA (Hagstromer et al. 2006). Additionally, IPAQ has been culturally adapted for the Finnish population (Craig et al. 2003). More specific calculations and assessments of the IPAQ have been described previously (Päivärinne et al. 2018). Participants were divided into tertiles based on their total LTPA: I (< 8 MET-h/week), II (8–28 MET-h/week), and III (> 28 MET-h/week) and total OPA: I (0 MET-h/week), II (< 64 MET-h/week), and III (≥ 64 MET-h/week).

### Work ability

Participant work ability was assessed by the Work Ability Index (WAI), which is a self-reported instrument that assesses work ability and diagnoses, symptoms, and sickness absence to measure the health and functional capacity via a single dimension of work ability (Ilmarinen et al. 2005). The WAI consists of seven items regarding both physical and psychological aspects of work ability. Scores range from 7 (lowest) to 49 (highest). The points of the WAI form the basis for determining the level of work ability according to the following scales: 7–27 (“poor” work ability), 28–36 (“moderate” work ability), 37–43 (“good” work ability) and 44–49 (“excellent” work ability) (Ilmarinen et al. 2005; Rautio and Michelsen 2014). The WAI has been demonstrated to be a valid instrument for assessing work ability (Lundin et al. 2017).

### Other variables

The participants’ occupational status were asked using the question “Are you currently…?” with response options of employee, clerical employee, superior, expert, department manager, senior manager or other. Diagnosed disorders were inquired through several different illnesses and were only taken into account if they were diagnosed by a doctor (accidental injury, musculoskeletal disease, circulatory disease, respiratory disease, or mental health disorders) (Ilmarinen et al. 2005; Rautio and Michelsen 2014). Daily alcohol consumption and frequency (weekly/monthly) over the past 12 months were asked using the question “During the past 12 months, how often have you had a drink containing alcohol?” with response options of never, 6–7 times a week, 4–5 times a week, 2–3 times a week, once a week, once a month or less than once a month (Aromaa and Koskinen 2004). The Finnish guidelines for high-risk alcohol consumption levels for healthy adult males are considered as more than 6 drinks at once and 23–24 drinks per week (Kauhanen et al. 1992). The numeric rating scale (NRS), which is a reliable and valid instrument for assessing pain were used to assess general pain, neck pain, upper limb pain, low back pain and lower limb pain, was used to assess general pain, lower back pain, lower limb pain, neck pain, or upper limb pain (Hawker et al. 2011). Score was scaled through 0–10, where 0 referred to “no pain” and 10 to “pain as bad as it could be” (McCaffery and Beebe 1993). Participants’ Body Mass Index (BMI) were calculated with a subject’s weight in kilograms divided by the square of the subject’s height in meters (kg/m^2^) (Khosla and Lowe 1967). Commonly used BMI ranges are underweight (below 18.5 kg/m^2^), normal weight (18.5–24.9 kg/m^2^), overweight (25.0–29.9 kg/m^2^) and obese (over 30.0 kg/m^2^) (World Health Organization Europe 2018).

### Statistical analyses

Data are presented as means with standard deviations (SD) or as counts with percentages. The statistical significance for the linearity across three LTPA and OPA levels (tertiles) was tested using analysis of variance (ANOVA) with appropriate contrast (orthogonal polynomial) and Cochran-Armitage test (Altman 1991). A possible nonlinear relationship between WAI and the standardized or continuous LTPA was assessed using three-knot-restricted cubic spline regression models. The length of the distribution of knots was located by Harrell’s default at the 10th, 50th, and 90th percentiles. Regression models included age, alcohol consumption, body mass index (BMI), employment years, and working class status as covariates. In case of violation of the assumptions (e.g., non-normality), a bootstrap-type method was used (10,000 replications) to estimate the standard error (Elfron and Tibshirani 1998); distribution of WAI index was skewed left. Effect sizes, eta-squared (*η*^2^), were calculated using the linear models (ANOVA). By convention, values of 0.01, 0.06, and 0.14 are deemed small, medium, and large effect sizes, respectively (Cohen 1988). The normality of variables was evaluated by the Shapiro–Wilk *W* test. Stata 15.0 (StataCorp LP; College Station, Texas, USA) statistical package was used for the analysis.

## Results

### Participant demographics

The sample consisted of 921 male participants who were under part-time or full-time employment. Demographic and clinical characteristics of the study participants according to LTPA (MET-h/week) are shown in Table 1. A small but statistically significant linear relationship was observed between several variables. Between subject age and LTPA; older subjects had lower LTPA MET-h/week scores (*P* = 0.009). Furthermore, linear relationships across MET tertiles were observed between the subjects’ BMI (*P* = 0.024), working class status (*P* = 0.040), employment years (*P* = 0.039), and presence of cardiovascular disorders (*P* = 0.001). Additionally, a linear relationship was observed between LTPA and weekly alcohol consumption (*P* = 0.005), general pain (*P* = 0.030), lower back pain (*P* = 0.036), neck pain (*P* = 0.001), upper limb pain (*P* = 0.001), and sick days during the last 12 months (*P* = 0.004).

**Table 1:** Demographic and clinical characteristics of the participants (*N* = 921) divided into tertiles according leisure-time physical activity (MET-h/week) levels

	I, (*n* = 304)	II, (*n* = 311)	III, (*n* = 306)	*P* value^a^
Age, mean (SD)	33.2 (6.3)	32.7 (5.9)	31.6 (6.4)	0.009
BMI, mean (SD)	26.4 (4.4)	26.2 (3.7)	25.7 (3.2)	0.024
Working class, *n* (%)				
Blue collar	216 (71.1)	185 (59.5)	193 (63.1)	0.040
Employment years, mean (SD)	7.1 (5.7)	7.4 (6.0)	6.2 (5.5)	0.039
Disorders, *n* (%)				
Accidents	29 (10)	34 (11)	36 (12)	0.38
Musculoskeletal disorders	60 (20)	67 (22)	56 (18)	0.66
Cardiovascular disorders	19 (6)	14 (5)	3 (1)	0.001
Lung disorders	11 (4)	14 (5)	14 (5)	0.56
Mental disorders	13 (4)	13 (4)	8 (3)	0.28
Alcohol consumption per week, mean (SD)^b^	7.7 (10.4)	6.9 (9.1)	5.7 (6.3)	0.005
Pain, NRS, mean (SD)	1.80 (2.05)	1.59 (1.96)	1.44 (1.86)	0.030
Low back pain	1.80 (2.19)	1.61 (2.04)	1.43 (2.01)	0.036
Lower limb pain	0.90 (1.62)	0.92 (1.74)	0.87 (1.48)	0.82
Neck pain	1.88 (2.19)	1.65 (2.11)	1.30 (1.76)	0.001
Upper limb pain	1.07 (1.97)	0.63 (1.46)	0.59 (1.38)	< 0.001
Sick days > 9 (last 12 months), *n* (%)	61 (20)	44 (14)	36 (12)	0.004
Occupational physical activity, *n* (%)				0.016
I (0 MET-h/week)	86 (28.3)	110 (35.4)	116 (37.9)	
II (< 64 MET-h/week)	99 (32.6)	107 (34.4)	90 (29.4)	
III (≥ 64 MET-h/week)	119 (39.1)	94 (30.2)	100 (32.7)	

*NRS* numeric rating scale

^a^*P* for linearity across MET tertiles. Count variables calculated using Cochrane–Armitage test and mean values by Anova

^b^Units per week

### Activity scores

**Fig. 1:**
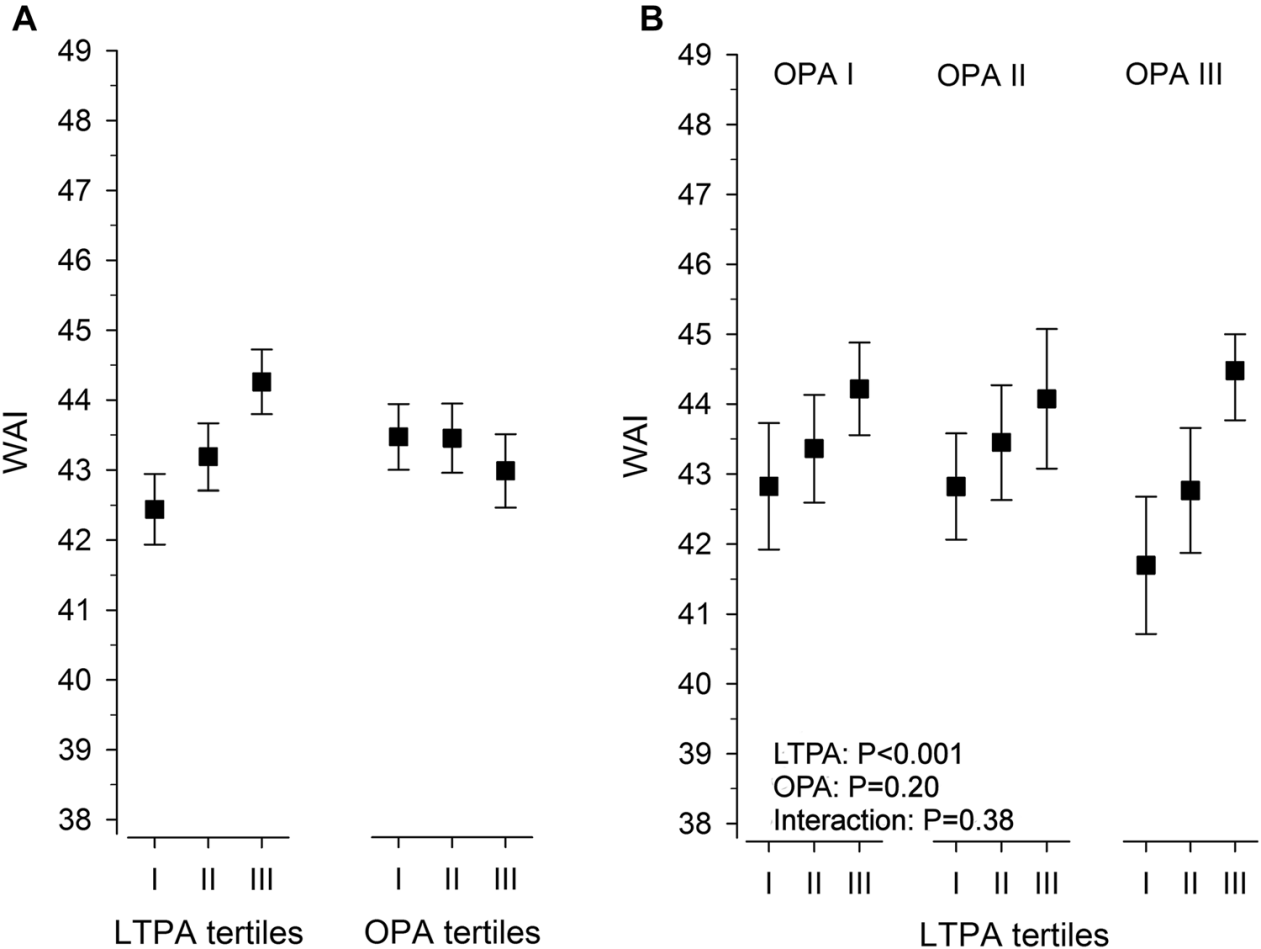
**a** Work ability index (WAI) in leisure-time physical activity (LTPA) tertiles and occupational physical activity (OPA) tertiles adjusted for age, alcohol consumption, BMI, employment years, and working class status. **b** Adjusted mean of WAI across the LTPA in each OPA tertile. Means with 95% confidence intervals are shown. *P* value shows linearity between the tertiles

In panel A of Fig. 1, age-, alcohol consumption-, BMI-, employment year-, and working class status-adjusted mean WAI are shown across the MET tertiles. After adjustment, LTPA associated with higher work ability (*P* < 0.001) [*η*^2^ = 0.03 (95% 0.01–0.05)], whereas different OPA levels did not associate with WAI. No interaction was observed between LTPA and OPA (Fig. 1b) tertiles [*η*^2^ = 0.00 (95% 0.00–0.01)].

### Relationship between activity levels and work ability

**Fig. 2:**
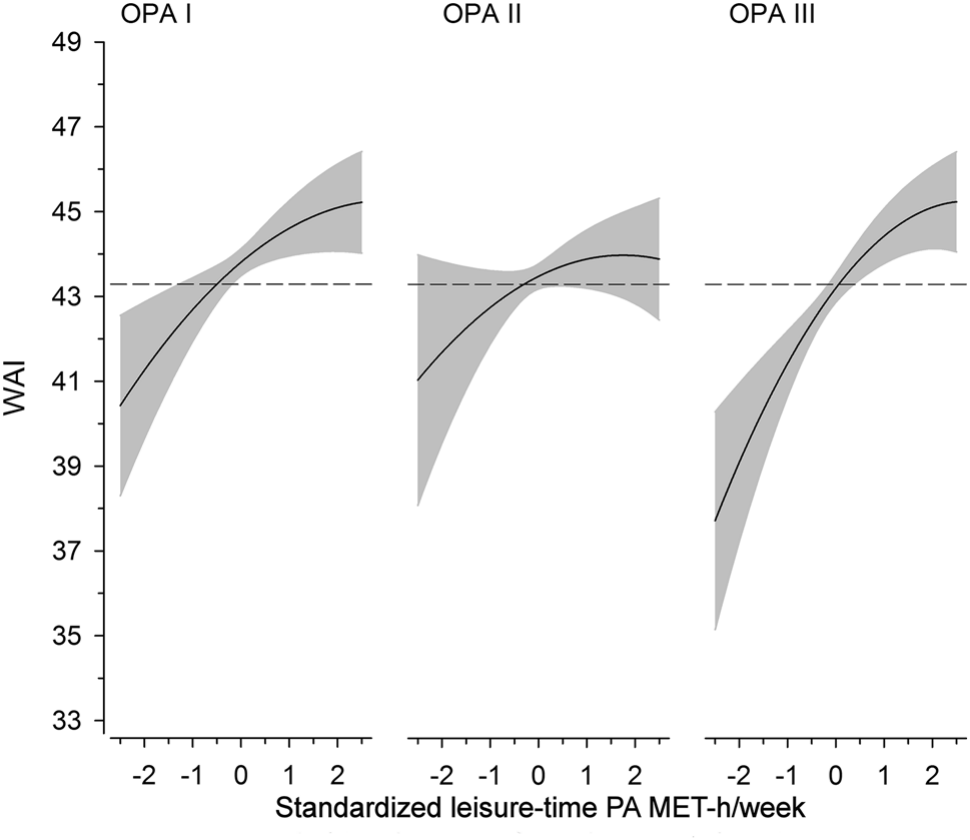
Relationship between standardized leisure-time physical activity (MET-h/week) and work ability index (WAI) in different occupational physical activity (OPA) tertiles. The curves were derived from a three-knot restricted cubic splines regression model. The model was adjusted for age, alcohol consumption, working class status, BMI, and employment years. The gray areas represent 95% confidence intervals. The dashed line indicates the mean value of all participants in WAI

Figure 2 illustrates the cubic spline regression of change in WAI in relation to 1 SD per standardized LTPA MET-h/week. In OPA tertile I, the WAI change per 1 SD was 0.93 (95% confidence interval [CI] 0.32–1.54; *P* = 0.003). In tertile II, the WAI change per 1 SD was 0.59 (95% [CI] − 0.14–1.32; *P* = 0.112). In tertile III, the WAI change per 1 SD was 1.51 (95% CI 0.88–2.14; *P* ≤ 0.001) after adjusting for age, alcohol consumption, working class status, BMI, and employment years.

**Fig. 3:**
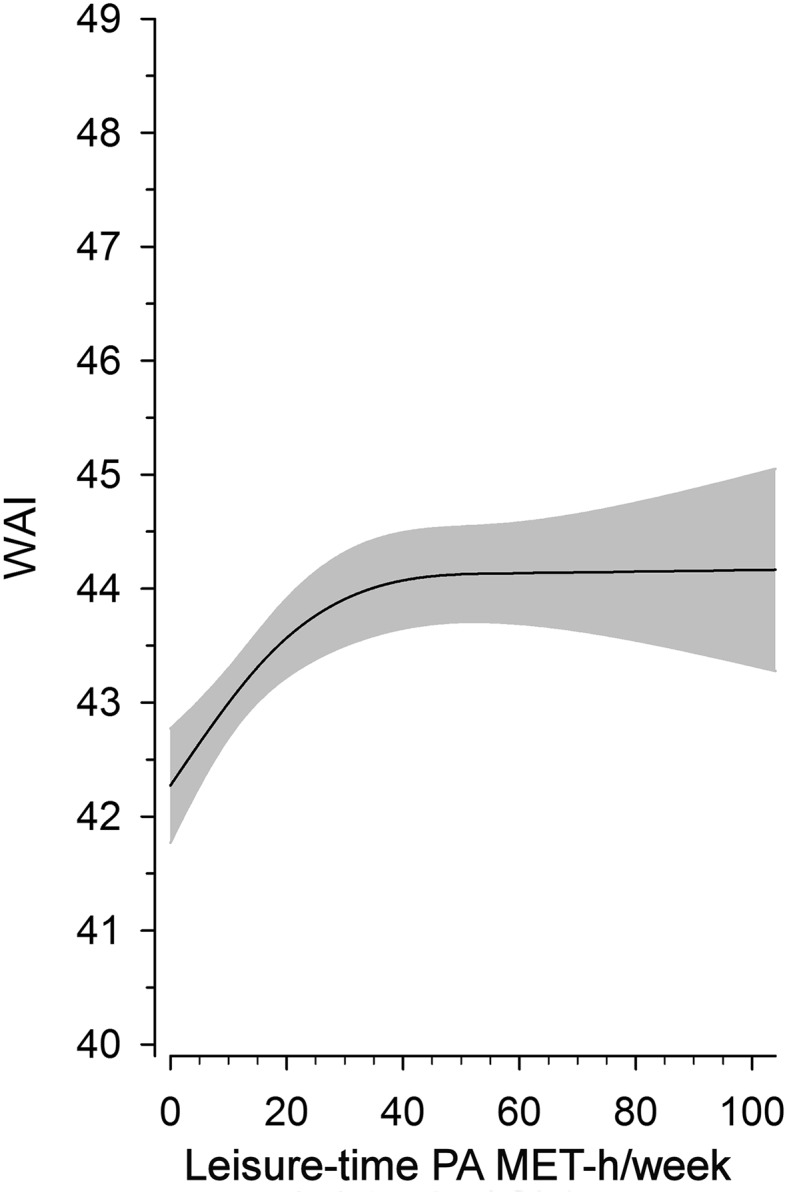
Relationship between continuous leisure-time physical activity (MET-h/week) and work ability index (WAI). The curves were derived from a three-knot restricted cubic splines regression model. The model was adjusted for age, alcohol consumption, working class status, BMI, and employment years. The gray area represents 95% confidence intervals

The cubic splines relationship between continuous LTPA and WAI is illustrated in Fig. 3. The regression curve showed a positive association between LTPA and WAI, particularly if weekly LTPA achieved approximately 40 MET-h/week.

## Discussion

The results presented here indicate that among a population of healthy male subjects between 20 and 40 years, self-reported LTPA (MET-h/week) associated with better work ability, particularly in subjects with more physically demanding jobs. Furthermore, the results indicate that when exposed to different OPA levels, higher LTPA levels were positively associated with WAI. More specifically, LTPA had a significantly greater relationship between WAI in subjects with physically demanding jobs than in subjects with more sedentary jobs. This association was independent of age, BMI, alcohol consumption, working class status, and employment years.

### Previous findings

Our findings are consistent with the systematic review of van den Berg et al. (2009), which describes a strong association between absence of vigorous LTPA and WAI in an adult population. Furthermore, LTPA has a positive relationship with WAI among a cohort of mostly female health care workers (Arvidson et al. 2013). There is also evidence from a randomized controlled study that physical exercise intervention improves work ability due to enhanced cardiorespiratory fitness (Kettunen et al. 2014). Moreover, evidence from a cross-sectional study revealed an association between high LTPA levels and better work ability in workers with physically demanding jobs (Calatayud et al. 2015). However, this study was conducted on middle-aged participants and a more heterogeneous population with different PA and work ability measurement methods. Information on combined associations of LTPA and work ability in different occupational physical demands is currently limited, particularly in young adult men.

### Interpretation of principal findings

In our study, the greater relationship between high LTPA and excessive OPA in work ability could be partially explained by the maintaining and improving effects of LTPA on cardiorespiratory fitness and health, which is normally included with voluntary movement and a sufficient amount of recovery time (Warburton et al. 2006). In contrast, OPA has been considered to have longer periods and lower intensity levels of maximal aerobic capacity, while elevating the 24-h heart rate and blood pressure, which may even impair cardiovascular health (Korshøj et al. 2015; Holtermann et al. 2018). In the present study, high levels of LTPA could also play a part in improving the responsiveness and adaptation of cardiovascular health and heart rate recovery (Lamberts et al. 2010). Since excessive periods of high OPA are reported to prolong high heart rates, high OPA and low cardiorespiratory fitness could cause cardiovascular strain that may lead to cardiovascular disorders. Higher cardiovascular fitness could possibly prevent these outcomes (Korshøj et al. 2015; Holtermann et al. 2016) and thus, appear in individuals with less illnesses, sick days and overall better work ability. Additionally, LTPA has been shown to decrease the risk for long-term sickness absence (LTSA), whereas OPA has been shown to increase the risk for LTSA (Holtermann et al. 2012). Moreover, LTPA has previously associated with fewer disability pensions because of musculoskeletal disorders (Fimland et al. 2015). Our study showed that the number of sick days linearly decreased with increasing LTPA MET tertiles.

As higher LTPA is associated with better physical and musculoskeletal fitness (Warburton et al. 2006), higher LTPA could be beneficial for those who are exposed to physical workloads including heavy lifting with static and constrained postures and activities, possibly impairing physical health functioning (Holtermann et al. 2012; Mänty et al. 2015). In our study, the positive association of LTPA could appear in the form of better self-assessed work ability, particularly in subjects with physically demanding jobs. On the other hand, it could also refer to better pain self-efficacy (Denison et al. 2007) strategies or other health-enhancing effects in relation to intensity, duration, or type of contraction caused by LTPA (Søgaard and Sjøgaard 2017). These effects could help more physically active participants to cope and adapt with the physical demands of high OPA (Holtermann et al. 2018). In our study, this could explain the inverse linear relationship across MET tertiles and the incidence of general pain, lower back pain, neck pain, and upper limb pain. In addition, individuals with low self-efficacy due to pain-concentrated disorders are reported to have a high probability of avoiding PA in their daily routines (Rejeski et al. 1998). The terminology of musculoskeletal disorders (MSD) is considered as a broad term covering any nonspecific disorder characterized by pain or decreased functioning (Søgaard and Sjøgaard 2017). Therefore, because of the possibly lower muscular health level in the lower PA group, lower WAI scores and higher pain incidence could indicate functional impairments in the future.

In addition, our study presented a smaller but significant relationship between lower OPA levels and LTPA. Nawrocka et al. (2017) found a relationship between achieving recommended LTPA levels and WAI among white-collar workers. In the present study, even though the majority of participants were blue-collar workers, our results indicated a similar relationship in the group that had the lowest OPA level. Other studies have shown that sedentary workplace behavior combined with low LTPA is associated with work-related fatigue, musculoskeletal pain, and a higher risk for heart failure or burnout (de Vries et al. 2017; Naczenski et al. 2017). In the present study, higher LTPA and musculoskeletal fitness could particularly reduce work-related pain in the lowest OPA group, which is caused by time spent in unnatural and static or standing positions (Coenen et al. 2016). Such pain reduction could relate to better WAI.

In the present study, the participants were men of 20–40 years who had good work ability and were expected to have many employment years in the course of their career. However, the risk of subsequent retirement due to disability and mortality associated with lack of PA is expected to increase in middle age (Lear et al. 2017). Our current study revealed an inverse linear relationship between LTPA and working years, meaning that subjects that had more years under employment were also less physically active. In addition, the positive relationship between work ability and LTPA, particularly at the highest OPA, could be considered noteworthy as we have previously observed that participants who recorded the greatest share of their PA from work (61%) had, in contrast, the lowest level of LTPA (16%) (Päivärinne et al. 2018). Our present study adds value to our previously reported importance of LTPA, particularly in occupations that are physically demanding. As reported previously, individual factors (such as aging and lack of PA) may be associated with midlife transition and an increased risk of retirement due to disability (Lahti et al. 2016).

### Relevance of findings

Our results provide important public health implications. Almost 30% of male employees in Finland in 2013 considered their work as physically demanding; this rate is presumably even higher in other countries (Kauppinen et al. 2013). Additionally, the present findings provide valuable information on the associations between LTPA and work ability at different OPA levels, particularly in young adult men. Despite the strong evidence for the benefits of PA, there is need for studies that assess the multidimensional behaviors of PA, such as LTPA and OPA and their association with work ability, which could help create strategies for preventing sickness absence or premature retirement due to disability while improving the health-enhancing effects of PA for workers.

### Strengths and limitations

Our study has several strengths. We used validated and widely employed questionnaires, such as the IPAQ long form to calculate LTPA and occupational PA along with the WAI. Our data were collected and analyzed from a random, homogeneous sample of young adult men, which allowed us to make reliable generalizations. We also recruited a relatively large sample of young Finnish adult men. However, there are limitations that should be taken into consideration. Due to the cross-sectional design of the study, the exposure and outcome are simultaneously assessed and, therefore, it is not possible to establish a true cause-and-effect relationship. Second, the subjective method of self-assessing LTPA and WAI may have resulted in reporting bias that could affect the outcome. Third, caution is advised when generalizing these results to women.

## Conclusion

In conclusion, LTPA has a positive association with work ability in young adult men. More specifically, the relationship between LTPA and WAI was significantly greater in physically demanding jobs than in more passive jobs. Even for the cross-sectional nature of the study, due to fairly large and homogeneous sample size and commonly used questionnaires, our results present a valid implication for the positive effects of LTPA in workers under higher work-related physical strain. The present findings provide valuable information on the associations between LTPA and work ability at different OPA levels, particularly in young adult men. More studies are warranted to support the findings that could help create strategies for preventing sickness absence or premature retirement while improving the health-enhancing effects of PA for workers.
